# Immune checkpoint inhibitor-induced hypopituitarism presenting as postoperative hyponatremia in a gastric cancer patient: a case report

**DOI:** 10.3389/fmed.2026.1783352

**Published:** 2026-06-16

**Authors:** Yufang Wang, Pei Zhang

**Affiliations:** 1 Department of Radiotherapy, Liaocheng Hospital Affiliated to Shandong First Medical University (Liaocheng People’s Hospital), Liaocheng, Shandong, China; 2 Department of Endocrinology, Liaocheng Hospital Affiliated to Shandong First Medical University (Liaocheng People’s Hospital), Liaocheng, Shandong, China

**Keywords:** gastric cancer, hypopituitarism, postoperative hyponatremia, secondary adrenal insufficiency, sintilimab

## Abstract

This report describes a gastric cancer patient who developed persistent postoperative hyponatremia following surgery, ultimately associated with immune checkpoint inhibitor-induced hypopituitarism with secondary adrenal insufficiency. Initial symptoms, including diarrhea, abdominal pain, and hyponatremia, were misattributed to common postoperative complications. Laboratory testing, including ACTH and cortisol levels, confirmed pituitary insufficiency. Steroid replacement therapy led to rapid symptom improvement and stabilization. This case highlights an underrecognized endocrine complication of immune checkpoint inhibitor therapy, emphasizing the need for early recognition, careful monitoring of endocrine function, and multidisciplinary management in postoperative cancer care.

## Introduction

Gastric cancer is a significant global health issue, with treatment often involving a combination of surgery, chemotherapy, and, in some cases, immunotherapy. While postoperative recovery typically focuses on common complications such as infection and wound healing, there are instances where less apparent, yet serious, issues arise that may complicate the recovery process. One such complication is the development of endocrine abnormalities, which can often go undiagnosed due to their subtle and nonspecific symptoms.

In recent years, immune checkpoint inhibitors (ICIs) have been increasingly used in the treatment of gastric cancer, offering promising outcomes, particularly in advanced stages. However, the use of ICIs has also been associated with a range of immune-related endocrinopathies, including hypopituitarism and secondary adrenal insufficiency, which can significantly affect patient outcomes if not recognized early. The occurrence of such endocrine complications, particularly in the postoperative setting, remains underreported in the literature and poses a diagnostic challenge, as symptoms overlap with more common postoperative complications.

This case report describes a patient who developed persistent hyponatremia after gastric cancer surgery, which was ultimately diagnosed as hypopituitarism with secondary adrenal insufficiency insufficiency that was associated with prior sintilimab therapy. The objective of this report is to raise awareness of this rare but serious complication, discuss the differential diagnosis, and emphasize the need for early endocrinological evaluation and multidisciplinary management in patients receiving immunotherapy who present with unexplained postoperative symptoms.

## Case history

A 73-year-old male patient, presented in March 2024, complaining of a one-month history of food obstruction, was seen at Liaocheng People’s Hospital Oncology Department. His ECOG performance status was 1, with a height of 170 cm and weight of 60 kg. Gastrointestinal endoscopy revealed a lesion at the gastroesophageal junction, and pathology confirmed adenocarcinoma. PD-L1 immunohistochemistry CPS score was approximately 15%. Chest and abdominal CT scans showed gastric cancer with multiple enlarged regional lymph nodes, along with benign prostatic hyperplasia. Blood routine and liver/kidney function tests were essentially normal. The final diagnosis was gastric cancer cT3N1M0 with benign prostatic hyperplasia.

## Treatment process

The patient began receiving neoadjuvant chemotherapy (Oxaliplatin 200 mg and Tegafur 60 mg, BID from days 1–14) in March 2024 for three cycles. During this treatment, the patient experienced mild gastrointestinal reactions and bone marrow suppression, but the food obstruction improved. On May 16, 2024, abdominal CT scans showed partial regression of the tumor, with an efficacy evaluation of partial response (PR). Given the patient’s and family’s concerns about surgery, the decision was made to add Sintilimab (an anti-PD-1 monoclonal antibody) to improve the treatment outcome. The patient underwent a fourth and fifth cycle of chemotherapy combined with immunotherapy on May 16 and June 17, 2024. On July 10, 2024, a follow-up blood test revealed worsening bone marrow suppression, which was managed with supportive treatments. The sixth cycle of chemotherapy was delayed to September 24, 2024, due to bone marrow suppression and fatigue, and was combined with a third dose of immunotherapy. On September 23, 2024, PET-CT showed that the gastric cancer remained active but had significantly reduced in size compared to May 16, 2024, with an efficacy evaluation of partial response (PR). Despite this, the patient, feeling fatigued, refused surgery and ultimately decided to discontinue further treatment (see Table 1).

**Table 1:** Preoperative diagnosis and treatment procedure.

Time	Treatment and evaluation	Relevant adverse reactions/assessments
March 8–April 24, 2024	Neoadjuvant chemotherapy: Oxaliplatin 200 mg (d1) + Tegafur 60 mg (BID, 1–14)	Mild gastrointestinal reactions (CTCAE grade 1), bone marrow suppression (grade 1)
May 16, 2024	CT scan evaluation: Tumor regression at the gastroesophageal junction	Food obstruction improvement, partial response (PR)
May 16, 2024; June 17, 2024	Chemotherapy + Immunotherapy: Oxaliplatin 200 mg (d1) + Tegafur 60 mg BID (d1-14) + Sintilimab 200 mg (d1)	Bone marrow suppression grade 2 (WBC 2.9 × 10^9/L, PLT 88 × 10^9/L); recovery after white blood cell and platelet support
September 23, 2024	PET-CT: Increased wall thickening and metabolism at the gastroesophageal junction	Tumor remains active but significantly reduced in size compared to May 16, 2024, partial response (PR)
September 24, 2024	Chemotherapy + Immunotherapy: Oxaliplatin 200 mg (d1) + Tegafur 60 mg BID (d1-14) + Sintilimab 200 mg (d1)	Continued management of bone marrow suppression

CTCAE, Common Terminology Criteria for Adverse Events, used to assess the severity of treatment-related side effects; BID, Twice a day; WBC, White Blood Cells; PLT, Platelets; PR, Partial Response, indicating a significant reduction in tumor size, but not complete disappearance.

Postoperative Abnormal Findings: On November 30, 2024, the patient was readmitted due to bloating, nausea, and loss of appetite after eating popcorn. Upon examination, the patient was conscious and in good spirits, with an ECOG score of 1. CT scans showed no evidence of significant disease progression. Blood routine was normal, but liver and kidney function tests revealed hyponatremia (128 mmol/L on December 4, 2024), which was attributed to recent poor appetite. High-sodium diet and symptomatic treatment were administered, and the patient consented to undergo radical total gastrectomy. On December 5, 2024, the patient successfully underwent laparoscopic radical total gastrectomy for gastric cancer. Postoperative pathology showed different types of adenocarcinoma at the gastroesophageal junction (moderately differentiated tubular adenocarcinoma, papillary adenocarcinoma, and mucinous adenocarcinoma), with local lymph node metastasis. Pathological results indicated mild tumor regression post-neoadjuvant chemotherapy. The ypTNM staging was yPT2N1MX. Postoperatively, the patient received intravenous nutritional support and pain pump therapy, with NRS scores ranging from 5 to 8. On the third day post-surgery, after passing gas, the patient started receiving enteral nutrition via a feeding tube. However, the patient developed diarrhea with watery stools, occurring more than ten times per day, without blood or pus. Enteral nutrition was discontinued and replaced with oral liquid diet combined with montmorillonite powder treatment. Diarrhea frequency decreased to 5–8 times per day, but the patient continued to experience intermittent abdominal pain, with an NRS score of 6–8, affecting their ability to eat. Concerned about anastomotic leakage, abdominal CT was performed, which showed no signs of leakage. Additionally, the patient experienced frequent nighttime urination, which disrupted sleep and caused daytime fatigue, irritability, and somnolence. Initial consideration was benign prostatic hyperplasia, and treatment with tamsulosin was initiated, but symptoms did not improve. Electrolyte tests were performed every 2 to 3days, and blood sodium levels ranged from 123 to 127 mmol/L. Initially, the hyponatremia was attributed to diarrhea and poor appetite, with high-concentration sodium supplementation provided. However, this did not resolve the issue. On December 20, 2024, the patient’s fatigue, irritability, and somnolence worsened, and repeat blood sodium levels dropped to 116 mmol/L. Clinical volume assessment revealed euvolemia, with no orthostatic hypotension, normal skin turgor, and absence of peripheral edema.

To evaluate the cause of hyponatremia, serum and urine osmolality were measured. The patient’s serum osmolality was 252 mOsm/kg (normal range 270–330 mOsm/kg), and urine osmolality was 597 mOsm/kg (normal range 600–1,000 mOsm/kg). Although serum osmolality was reduced, urine osmolality was not inappropriately elevated given the degree of hyponatremia.

Given the patient’s history of immunotherapy with sintilimab and persistent hyponatremia unresponsive to sodium supplementation, pituitary insufficiency was suspected. Endocrinological evaluation was therefore performed. The results showed an ACTH level of 8.01 pg./mL (normal range 7.2–63.3 pg./mL) and cortisol levels of 1.14 μg/dL, 1.31 μg/dL, and 1.44 μg/dL at 1:00 a.m., 8:00 a.m., and 4:00 p.m., respectively, all below the normal range (6.7–22.6 μg/dL), as summarized in Table 2. Thyroid function and other hormone axes, including growth hormone and gonadal hormones, were within normal limits. Based on these findings, the patient was diagnosed with hypopituitarism resulting in secondary adrenal insufficiency. He subsequently started steroid replacement therapy with prednisone (5 mg in the morning and 2.5 mg in the evening). Blood sodium levels normalized rapidly, and his symptoms of fatigue, diarrhea, and abdominal pain resolved quickly. This prompt clinical response further supported the diagnosis and effectively ruled out SIADH as the primary cause of hyponatremia.

**Table 2:** Summarizes the key hormonal findings.

Hormone	Result	Normal range
ACTH	8.01 pg./mL	7.2–63.3 pg./mL
Cortisol (1:00 a.m.)	1.14 μg/dL	6.7–22.6 μg/dL
Cortisol (8:00 a.m.)	1.31 μg/dL	6.7–22.6 μg/dL
Cortisol (4:00 p.m.)	1.44 μg/dL	6.7–22.6 μg/dL

## Discussion

### Differential diagnosis

The initial differential diagnosis for this patient’s hyponatremia included common postoperative complications (diarrhea and poor intake), syndrome of inappropriate antidiuretic hormone secretion (SIADH), primary adrenal insufficiency, and hypothyroidism. Although serum osmolality was reduced (252 mOsm/kg), urine osmolality was not inappropriately elevated (597 mOsm/kg) given the degree of hyponatremia, making SIADH unlikely. Normal thyroid function tests excluded hypothyroidism. The finding of low-normal ACTH (8.01 pg./mL) with severely low cortisol levels (as low as 1.14 μg/dL) pointed to secondary adrenal insufficiency due to hypopituitarism, rather than primary adrenal insufficiency (in which ACTH is typically elevated). Metastatic disease to the pituitary was considered unlikely because a PET-CT performed on December 4, 2024, showed no evidence of brain or sellar region metastases. Postoperative stress alone is an unlikely cause of persistent, severe hyponatremia unresponsive to sodium supplementation, as postoperative cortisol levels typically rise rather than fall. The patient’s critically low cortisol levels (as low as 1.14 μg/dL) are incompatible with a normal stress response.

### Literature review

Currently, the pathogenesis of hypopituitarism associated with immune checkpoint inhibitors (ICIs) is not fully understood. The mechanisms of CTLA-4 inhibitors and PD-1/PD-L1 inhibitors leading to adrenal insufficiency may differ, but both involve immune reactions induced by immune checkpoint inhibitors that cause pituitary inflammation (1, 2). According to reports, the incidence of pituitary inflammation associated with ICIs is higher with CTLA-4 inhibitors than with PD-1/PD-L1 inhibitors. Additionally, the incidence is higher when ICIs are used in combination compared to monotherapy. In monotherapy, the incidence of pituitary inflammation is reported to be 3.2% for CTLA-4 inhibitors, 0.4% for PD-1 inhibitors, and less than 0.1% for PD-L1 inhibitors (3). In the sintilimab package insert, pituitary inflammation occurred in 0.5% of patients, with a median onset of 197 days. In this case, hyponatremia was already present before surgery (128 mmol/L on December 4, 2024), suggesting that the endocrinopathy may have developed before the postoperative period.

Clinically, pituitary inflammation manifests as hormone deficiency symptoms and pituitary enlargement caused by inflammation, leading to mass effect such as headaches, fatigue, and general weakness. Other possible symptoms include nausea, anorexia, diarrhea, weight loss, mental disorders, hypotension, hypoglycemia, polyuria, chills, and sexual dysfunction (4, 5).

### Clinical implications

This case illustrates the diagnostic challenge posed by ICI-induced hypopituitarism in the postoperative setting. Symptoms easily mimic common postoperative issues, leading to delayed diagnosis. Persistent hyponatremia unresponsive to sodium supplementation should prompt endocrinological evaluation in patients with a history of ICI therapy. According to the 2020 Chinese Society of Endocrinology Expert Consensus on Immune-related Endocrinopathies Caused by Immune Checkpoint Inhibitors (6), it is recommended that pituitary-targeted hormone levels be monitored monthly during the first 6 months of immunotherapy; every 3 months during the subsequent 6–12 months; and annually after 2 years. When acute adrenal insufficiency caused by ICIs is suspected, ICI treatment should be suspended, and cortisol and ACTH tests should be performed immediately, followed by consultation with the endocrinology department and the initiation of hydrocortisone treatment before other hormone replacement therapies (7, 8). In alignment with the Chinese expert consensus, multiple international guidelines have been published for the management of immune-related endocrinopathies. The American Society of Clinical Oncology (ASCO) and the European Society for Medical Oncology (ESMO) have established comprehensive recommendations for the diagnosis, grading, and treatment of irAEs. A key principle emphasized across these guidelines is that endocrine irAEs, including hypopituitarism and secondary adrenal insufficiency, should be managed with physiologic hormone replacement rather than high-dose corticosteroids, which are reserved for other irAEs. These international guidelines also recommend baseline endocrine testing before ICI initiation and periodic monitoring of morning cortisol and thyroid function during treatment.

### Limitations

Pituitary MRI was not performed because the patient declined the examination, which is an important limitation of this case. Pituitary MRI is the gold standard for identifying structural abnormalities of the pituitary gland, including inflammatory enlargement (hypophysitis) or metastatic lesions. The absence of this imaging study introduces uncertainty regarding the potential structural pathology of the pituitary gland. However, a PET-CT performed before surgery (December 4, 2024) showed no evidence of brain metastasis, and the rapid clinical response to steroid replacement therapy supports the diagnosis of immune-related hypopituitarism. Nevertheless, future cases should strongly encourage pituitary MRI to achieve a more accurate diagnosis and guide appropriate management.

In addition to the absence of pituitary MRI, dynamic endocrine testing (such as the ACTH stimulation test) was not performed. The patient’s baseline cortisol levels were critically low (as low as 1.14 μg/dL, normal 6.7–22.6 μg/dL), which alone is strongly suggestive of adrenal insufficiency. In the acute setting with life-threatening hyponatremia (116 mmol/L) and neurological symptoms, immediate steroid replacement was clinically mandated, and dynamic testing would have delayed necessary treatment. Furthermore, the rapid clinical and biochemical response to glucocorticoid therapy provides strong confirmatory evidence for the diagnosis of secondary adrenal insufficiency.

## Conclusion

This case demonstrates that sintilimab, an anti-PD-1 antibody, can induce hypopituitarism and secondary adrenal insufficiency, presenting as persistent postoperative hyponatremia. These symptoms may be easily mistaken for common postoperative complications in gastric cancer patients. Early recognition and timely hormone replacement therapy are critical to prevent life-threatening adrenal crises and improve patient outcomes. Further studies are needed to establish standardized monitoring protocols for ICI-induced endocrinopathies.

## Data Availability

The original contributions presented in the study are included in the article/supplementary material, further inquiries can be directed to the corresponding author.
